# Fibroblasts from HPV-negative oropharynx squamous cell carcinomas stimulate the release of osteopontin from cancer cells via the release of IL-6

**DOI:** 10.3389/froh.2024.1390081

**Published:** 2024-05-13

**Authors:** Naeima Yahia Hendawi, Hannah L. Crane, Hisham Mehanna, Robert Bolt, Daniel W. Lambert, Keith D. Hunter

**Affiliations:** ^1^ Academic Unit of Oral Medicine and Pathology, School of Clinical Dentistry, University of Sheffield, Sheffield, United Kingdom; ^2^ Faculty of Dentistry, University of Benghazi, Benghazi, Libya; ^3^ Institute of Cancer and Genomic Sciences, University of Birmingham, Birmingham, United Kingdom; ^4^ Liverpool Head and Neck Centre, Molecular and Clinical Cancer Medicine, University of Liverpool, Liverpool, United Kingdom

**Keywords:** oropharynx, squamous cell carcinoma, human papilloma virus, tumour microenvironment, osteopontin, SPP1, IL-6

## Abstract

**Introduction:**

HPV-associated oropharyngeal squamous cell carcinoma (OPSCC) shows distinct biological and clinical behaviour when compared to HPV-negative OPSCC. The overall role of the tumour microenvironment (TME) in head and neck cancer progression and metastasis has been studied intensively, but differences in HPV-negative and HPV-positive OPSCCs are less understood.

**Objective:**

To investigate the role of cancer-associated fibroblasts (CAFs) and the functional interactions of normal tonsil fibroblasts (NTFs) and OP CAFs with HPV+ and HPV− OPSCC cells and explore novel candidates in tumour-fibroblast crosstalk.

**Materials and methods:**

A retrospective cohort of 143 primary OPSCCs was characterised using HPV16/18 RNAScope assay, p16 IHC and ɑ-SMA. Four OPSCC, three NTF and 2 new OPSCC CAF cultures were used to assess the cytokine-based interactions using cytokine arrays on conditioned media (CM), followed by co-culture approaches to identify the role of individual cell types and the role of OPN (SPP1) and IL-6 in SCC/fibroblast communication.

**Results:**

HPV status was associated with better overall survival. Although ɑ-SMA expression was observed in both OPSCC subtypes, it provided survival stratification only in the HPV−positive group (Log-Rank *p* = 0.02). Three normal tonsillar fibroblast cultures (NTFs) were characterised by induction of myofibroblastic and senescent phenotypes with similar reactivity to our published NOF phenotype. The OPSCC-derived CAF cultures were characterised and their baseline myofibroblastic and senescence phenotypes varied. Cytokine array analysis of CM to identify novel candidates in the crosstalk between OPSCC tumour cells and NTFs/CAFs identified differences in the cytokine profiles on comparison of HPV+ and HPV− OPSCC cells. Osteopontin (OPN/SPP1) was identified, particularly in HPV-negative OPSCC cell analyses. We have demonstrated that OPN was produced by the OPSCC cells and revealed an associated upregulation of IL-6 in fibroblasts. Treatment of NTFs with rOPN showed alteration in phenotype, including increased contraction and IL-6 production. Antibody-mediated inhibition of CD44v6 attenuated the production of IL-6 by OPN in NTFs.

**Conclusion:**

This investigation with OPSCC fibroblasts provides novel insights into the role of CAFs in OPSCC mediated by IL-6 stimulated release of OPN from HPV negative OPSCC cells. The details of HPV-positive SCC cell/fibroblast cytokine crosstalk remain elusive.

## Introduction

The past few decades have seen a marked increase in squamous cell carcinomas (SCCs) of the oropharynx. In the UK, this has included increases in the incidence of both HPV-associated SCC and conventional SCC (1, 2). These subtypes bring differing clinical and molecular features with associated differences in survival (3). Given the superior prognosis of HPV-driven OPSCC there is wide interest in treatment de-intensification programmes, but initial results of these clinical trials have been disappointing (4, 5). In this context, it is an interesting question if patient selection based on HPV-status alone is too simplistic and may require a deeper understanding of the biology of both the epithelial component and the tumour microenvironment (TME) to support more appropriate selection of patients for such de-intensification approaches.

The tumour microenvironment comprises a rich community of cellular and non-cellular components which have been shown to play a key role in the development and progression of many malignant neoplasms (6). In oral cavity SCC, the presence of an extensive, activated myofibroblast population in the TME (as measured by αSMA expression), is a better predictor of poor clinical outcomes than many of the traditional clinical staging tools (7).

Fibroblasts in the TME of solid tumours, frequently termed cancer associated fibroblasts (CAFs) are a variably prominent component which exert profound influences on tumour behaviour. CAFs have shown the capability to enhance tumour growth, local invasion, and metastasis (8, 9) and are correlated with poor clinical outcome in several tumours (10–13). Single cell sequencing of the TME in HNSCC has confirmed that CAFs are not a homogeneous group; several sub-populations have been identified, some of which have been associated with poor prognosis, whilst others with a more favourable prognosis (14, 15). Even in those fibroblasts which express high levels of *α*SMA, there is significant heterogeneity, as these may by induced by several stimuli, including TGF-β1 and cellular senescence (16).

Although well-established for OSCC, the role that the CAFs play in OPSCC has been less thoroughly investigated. Recent RNASeq analysis of a cohort of OPSCCs indicated that the TME in HPV-positive and negative OPSCC was differentially enriched: HPV-negative dominated by fibroblasts and capillary endothelial cells, whereas the HPV-OPSCC TME was enriched for adaptive immune cells (17), suggesting fundamental differences in the TME exist. Similar differences in the fibroblast content have also been noted on multiplex image cytometry (18).

We have previously reported differences in the interaction of HPV-positive and HPV-negative OPSCC cell lines with fibroblasts in various 2D and 3D models (19). HPV-negative cell lines were able to stimulate normal oral fibroblasts to produce a secretome which enhanced cancer cell migration and invasion, whilst HPV-positive cells did not, this at least in part due to the action of HGF. These interesting observations highlight the need for further investigation and clinical validation of fibroblast function in OPSCC model systems and in tissues. In this study we extend our investigation of the fibroblast component of the TME in OPSCC, focussing on its relation to clinical outcomes and how epithelial cell HPV-status is related to the interactions of tonsil-derived fibroblasts and OPSCC cells.

## Methods

### Cell lines and culture conditions

OPSCC cell lines UD SCC02, UPCI SCC072, UPCI SCC089 and UPCI SCC090 were used in this project: the UPCI cell lines were received under MTA from Prof. S. Gollin, University of Pittsburgh School of Public Health, Pittsburgh, PA. The HPV status was confirmed as previously described (19). Short tandem repeat profiling was undertaken to confirm cell line authenticity. A summary of cell lines used is given in Supplementary Table S1.

Fibroblast cultures were derived from normal tonsil and OPSCC explant cultures respectively, (NTF06, NTF10, NTF322, CAF1, CAF2) as described previously (20), using tissue retrieved from patients during ENT surgery, with written, informed consent, under ethical approval (09/H1308/66 and 13/NS/0120: Supplementary Table S2). The authors are grateful to Dr Helen Colley for access to the NTF cultures). Both fibroblasts and OPSCC cell lines were cultured in Dulbecco's Modified Eagle's Medium (DMEM) supplemented with 10% fetal calf serum, with 2 mM L-glutamine and 50 IU penicillin and 50 μg ml^−1^ streptomycin and incubated under standard conditions (5% CO2, 37°C). All cell cultures were subject to regular mycoplasma testing.

### CAF growth rates

CAF1 and CAF2 were seeded at a density of 5,000 cell/ml in a 6 well-plate with 2 ml growth media for 6 days. At daily intervals, cells from one well of the 6 well-plates were detached and counted giving the total count/ml of cells.

### Fibroblast culture treatments

TGF-β1: Cells were plated into 6-well plates (Greiner Bio-one, UK) at a density of 1.5 × 10^5^ per well with 2 ml of growth media and incubated overnight at 37°C. The next day, the media was aspirated, and cells were washed with PBS x2 and serum-starved overnight using DMEM serum-free media. On the third day, 2 ml of DMEM serum-free medium containing 5 ng/ml rh-TGF-β1 (Sigma-Aldrich, UK) was added to each well. The experimental control wells were incubated with 1 ml serum-free medium. Cells were incubated with 5 ng/ml TGF-β1 (R&D Systems, USA) for 24, 48 or 72 h. At the end of the experiment, cells were pelleted for downstream RNA/protein analysis and the media was aspirated, centrifuged, and the supernatant was stored at −20 °C.

**H_2_O_2_:** Early-passage NTF322, NTF6 and NTF10 cells were grown in 75 cm^2^ flasks to 70% confluence. Cells were then washed with PBS and incubated with 500 µM of H_2_O_2_ (Sigma-Aldrich, UK) in serum-free media for 2 h. Negative control cells were incubated with serum-free media alone. Cells were then washed with PBS x3 and incubated with growth media for 15 days. Cells were split on day 8. At the end of the experiment, the media were collected, centrifuged at 3,000 rpm for 5 min and stored at −20 °C. Cells were counted and the culture viability percentage was recorded. 4 × 10^4^ cells were seeded in a 12-well plate, prepared for Senescence-Associated β-galactosidase Assay (SA-β-gal) staining. The rest of the suspension was centrifuged, and the formed pellet was collected and stored at −80 °C for further analysis.

**Recombinant Osteopontin (rOPN):** Fibroblasts were cultured in a T75 cm^2^ flask in growth media to 70% confluence. Cells were washed with PBS x3 and serum-starved overnight. On the third day, cells were incubated with 10 ml of serum-free medium containing 180 ng/ml of rh-OPN (R&D systems, USA) for 48 h. The experimental control was incubated with 10 ml serum-free media only. At the end of the experiment, the media were aspirated, centrifuged, and stored at −20 °C for IL-6 ELISA analysis. Cells were harvested and counted to normalise ELISA analysis results then cells were collected for further IL-6 gene evaluation.

**Anti-CD44v6 antibody:** NTF322 and NTF10 were cultured in T75 cm^2^ flask with growth media up to 70% confluence. Cells were incubated for 1 h with serum-free media supplemented with 5 µg/ml of CD44var (v6) Monoclonal Antibody (VFF-18) (eBioscience™ Catalogue: BMS125). The experimental control was incubated with serum-free media. 6.5 ml of cell line conditioned media from SCC89, SCC72 and SCC2 was thawed and supplemented with 1.5 ml of fresh growth media. After 1 h of CD44 blocking, rOPN-containing or control CM was added to NTFs and incubated for 24 h. The next day, media were collected, centrifuged, and stored at −20 °C. Fibroblasts were harvested, counted, and collected as a cell pellet for further analysis.

### Collagen gel contraction assay

Following the protocol of the collagen gel contraction kit (Cell Bio-Lab Inc, CBA-201), NTFs were harvested and resuspended in growth medium at 2 × 10^4^ cells/ml. A collagen lattice was prepared by mixing 2 parts of cell suspension and 8 parts of cold collagen gel working solution. From this mixture, 0.5 ml was pipetted in a well in a 24-well plate and incubated for an hour at 37 °C. After collagen polymerization, 1 ml of growth medium was added, and the plate incubated for 24 h at 37 °C. The next day, the cells were serum-starved for a further 24 h, then were treated with 5 ng/ml TGF-β1 or 180 ng/ml OPN before releasing the stressed gel matrix from the plate using a sterile spatula. The gels were then incubated for 72 h (TGF-β1 stimulation experiment) or 48 h (rOPN stimulation experiment). Photographs of the gel disc were taken at 24, 48 and 72 h. The contractility of the gels was calculated by measuring the distance between the collage disc border and the plate well using ImageJ (NIH).

### Collection of conditioned media

Cell lines were grown to 70% confluence in T75 flasks, washed in PBS and incubated with fresh normal media for 24 h. Conditioned medium (CM) was then retrieved, centrifuged at 3,000 rpm for 5 min to remove cell debris and stored at −20 °C. The remaining cells were counted, and CM normalized to a concentration of 3 × 10^6 ^cells/ml.

Tonsil fibroblast cultures (normal and CAF) were grown to confluence in T75 flasks, washed in PBS and incubated with either OPSCC cell line CM or normal media control for 24 h. This media was collected (M1) and flasks were washed in PBS and incubated with fresh normal media for a further 24 h to collect a “stimulated” fibroblast CM (M2). CM was retrieved, centrifuged at 3,000 rpm for 5 min and stored at −20 °C. The remaining cells were counted to confirm an equal number of cells in each experiment post-stimulation.

OPSCC cell lines were exposed to unstimulated fibroblast CM collected in a similar manner to that described above.

### Transwell co-culture

Cancer cells (3 × 10^5^) were seeded in 1.5 ml of growth media on cell culture inserts containing a 0.45 μm plastic membrane filter and placed on a 6-well plate filled with 2.6 ml of growth media. Cells were allowed to adhere overnight. The filters were conditioned before cell seeding to enhance the cellular adhesion by preincubation for a minimum of 1 h at 37 °C. 50,000 cells of NTF 3, NTF 10, CAF1 or CAF2 were plated in 6-well plate in 2 ml growth media and incubated for 24 h The experimental controls were plated in the same way but omitting the fibroblasts or cancer cells. The next day, 1.5 ml fresh growth media was added to the inserts and 2.6 fresh growth media was added to the fibroblasts in 6-well plate, then the cell culture inserts were inserted into the wells carefully to avoid air bubble formation. The co-culture system was maintained for 48 h at 37 °C. At the end of the experiment, the media were aspirated and centrifuged at 3,000 rpm for 5 min and stored immediately at −20 °C. Cancer cells and fibroblasts were detached from the culture system, counted, and stored separately as pellets at −80 °C.

### Cytokine array

Serum-free conditioned media were collected from cell lines and stimulated fibroblasts as described above and soluble factors analysed by cytokine array Proteome Profiler Human XL Oncology Array kit (R&D systems: ARY026) following the manufacturer's protocol. This kit provides relative expression levels for 84 human cancer-related proteins. Developed films were scanned at high resolution and analysed using ImageJ Studio software (Version 5.2; LI-COR, Inc, USA). Densitometry data were then normalised to the positive control spot (reference spots).

### qPCR

Total RNA was extracted from cell pellets using an Isolate II RNA Mini Kit (Bioline), following the manufacturer's instructions. RNA was quantified using a Nanodrop 1,000 Spectrophotometer (Thermo Fisher Scientific). Five hundred nanograms of isolated RNA was reverse transcribed using a High-Capacity cDNA Reverse Transcription Kit (Applied Biosystems), following the manufacturer's protocol using a Peltier thermal cycler (MJ Research). cDNA was then stored at −20 °C.

Gene expression was quantified using a Rotor-gene Q real-time PCR cycler (Qiagen) with SYBR green or TaqMan chemistry. Quantification was achieved using delta CT values normalized to either U6 or B2M. Each reaction was performed in triplicate. The standard thermal cycle settings for a reaction consisted of 40 cycles (each 95 °C for 10 s, 60 °C for 15 s and 72 °C for 20 s), including a melt curve analysis (when using SYBR green).

Taqman (inventoried, Thermo Fisher Scientific, UK) and SYBR green primers (designed in-house, purchased from Sigma-Aldrich) were used, as seen in Supplementary Table S3.

### Western blot

Cell lines were grown to approximately 70% confluence, washed in PBS. On completion of each respective incubation period (as above), flasks were washed in cold TBS and detached using cell dissociation solution at 4 °C on a rocking machine for 10 min. Cells were centrifuged at 1,000 rpm for 5 min. Cell pellets were lysed on ice in 50 mM Tris–HCL pH 7.4, 250 mM NaCl, 5 mM EDTA, 0.3% Triton X-100 and EDTA-free protease inhibitor cocktail (Roche, Germany).

Lysates were boiled for 5 min in an equal volume of sample buffer and separated using 4%–15% polyacrylamide pre-cast gels (Mini-Protean TGX, Bio-Rad, CA). Separated samples were transferred to a nitrocellulose membrane (Amersham Hybond ECL, GE Healthcare, Chicago), then blocked in 5% skimmed milk in Tris-buffered saline (pH 7.4) with 0.1% Tween-20. This was followed by incubation with primary antibody (Supplementary Table S4) or anti-β actin control (1:3,000) in 5% bovine serum albumin/TBST overnight at 4 °C. Membranes were then incubated with horseradish peroxidase conjugated secondary antibody at a dilution of 1:10,000 for 45 min and imaged using ECL reagent (GE Healthcare).

### Immunofluorescence

An 8-well slide chamber (Starstedt, GER) was used to culture the cells for immunofluorescence analysis. 20,000 cells were seeded in each chamber with 800 μl of growth media. Chambers were incubated for 24 h at 37 °C in a humidified incubator with 5% CO2. After treatment with TGF-β1 or OPN, cells were fixed using the recommended reagent according to the manufacturer's instructions This was followed by another incubation with the permeabilization reagent if needed. After that, cells were blocked by the appropriate blocking on a shaker for 15 mins. 200 μl of the diluted primary antibody was added, including the experiment negative control, and incubated for 1.5 h (Supplementary Table S4). Cells were then washed twice with PBS and incubated with the secondary antibody for 1 h. The antibody control chamber was incubated with 200 μl of secondary antibody only. The chambers were washed x3 with PBS and mounted with a coverslip containing a drop of Prolong diamond antifade mountant (DAPI, Thermo Fisher P36966). The slide was placed in a slide box at 4 °C for 24 h. Images were captured using a Ziess Axioplan2 fluorescence light microscope (Carl Zeiss, UK). Staining intensity was quantified using Image J software quantifying the green colour only in fields with equal numbers of cells.

### IL-6 ELISA

Secreted IL-6 was quantified using the BD OptEIA (BD Biosciences, San Jose, USA) kit for human IL-6 detection. The procedure was performed following the manufacturer's recommendation using 96-well plates. Absorbance was read at 450 nm within 30 min of adding the stop solution. Wavelength correction was calculated by subtracting the absorbance at 570 nm from the absorbance at 450 nm.

### Senescence associated β-galactosidase staining

Twenty thousand cells per well were seeded in a 12-well plate and left to adhere overnight. Senescence associated β-galactosidase activity was assessed using a senescence detection kit (ab65351, Abcam) following the manufacturer's recommendations.

### Tissue microarray (TMA) cohort and construction

This study utilised patient samples and associated clinical data within the period of primary diagnosis 2002–2012. Patients were selected using the Oncology databases and cross-referenced against the histopathology archive at Sheffield Teaching Hospitals NHS Trust. Patients were included in the study if there was sufficient tissue remaining after diagnosis for TMA generation and if there was a reasonably complete dataset of associated clinicopathological information. All biopsy material was obtained before commencement of anti-cancer treatment. Paraffin-wax embedded tissue samples were retrieved and collated in a blinded fashion with respect to HPV status. Clinical data regarding tumour recurrence, clinical status and overall survival were retrieved from the patient's medical files at the Sheffield Teaching Hospital NHS Foundation Trust, UK. Overall survival was determined by the difference between the date of treatment and either the date of death due to the tumour or last follow-up. The study was conducted with National Research Ethical Committee approval (UK 12/LO/2018).

TMAs were constructed by selecting tumour regions displaying more than 70% cellularity with minimal necrosis and marked on haematoxylin and eosin-stained sections. The TMAs were constructed using a Beecher tissue arrayer with 3 × 1.0 mm diameter cores from each tissue block arranged at mapped locations into recipient paraffin blocks. TMAs were sectioned (5 μm) by microtome onto Superfrost™ adhesive glass slides (ThermoFisher Scientific).

An additional cohort of 96 cases was added, which had formed part of the PREDICTR study cohort (21). These were received from the University of Birmingham under MTA. An overall summary of the whole cohort is presented in Table 1.

**Table 1:** OPSCC clinical cohort details. Regarding treatment, all patients were treated with curative intent, and the treatment noted in the primary modality. Some patients underwent multi-modality treatment.

	HPV- positive	HPV- negative	Total	*p*-value
Number of cases	87	56	143	-
Age at diagnosis				
<45	9	6	15	0.71
45–55	31	15	46	
56–65	33	24	57	
>65	14	11	25	
Mean	56.1	56.9		0.91
Median	55	57.5		
Sex				
Male	65	39	104	0.65
Female	22	17	39	
Smoking^a^				
Never	38	7	45	<0.001
Former	20	15	35	
Current	20	29	49	
T-stage				
T1/T2	56	28	84	0.07
T3/T4	31	28	59	
N-stage				
N0/N2a	30	17	47	0.07
N2b/N3	57	39	96	
TNM staging				
III	30	30	60	0.02
IV	57	26	83	
Treatment				
Surgery	31	38	69	<0.001
Chemo-radiotherapy/ Radiotherapy	56	18	74	
Recurrence				
Yes	9	14	23	0.03
No	78	42	120	
Survival				0.002
Alive	14	21	35	
Dead	73	35	108	

^a^ Note that for smoking status, this data is incomplete.

### Immunohistochemistry

Immunohistochemistry for p16 and *α*SMA was performed on 4 µm formalin-fixed paraffin-embedded TMA sections. Human skin sections were used as positive controls. Antigen retrieval was performed with heat-induced epitope retrieval in sodium citrate buffer (10 mM sodium citrate, 0.05% Tween 20, pH 6.0). The primary antibody used are noted in Supplementary Table S4, and samples were incubated overnight at 4 °C. After secondary antibody incubation, staining was visualized using a Vectastain ABC Kit (Vector Laboratories) with 3,3′-diaminobenzidine substrate and a haematoxylin counterstain. Staining was assessed as follows:

• p16 (E6H4 CINTECH, using standard diagnostic p16 protocol). Expression was assessed semi-quantitatively, using H-score, to a maximum score of 300. Staining intensity (0–3) and % of cells at each stating level is recorded.
• αSMA (1:100). Staining was assessed as “high” or “low” using published assessment criteria (21). In general, low cases had focal <50% of stroma expressing *α*SMA, whilst high is >50%.

### RNAScope ISH

HR-HPV16, 18 E6 and E7 RNA ISH was performed with the RNAscope assay (Advanced Cell Diagnostics, Newark, CA, USA), according to the manufacturer's instructions. The following scoring system was followed for HPV RNA ISH slide assessment: 0: No staining or <1 dot every 10 cells; ×40 magnification. 1: 1–3 dots/cell; ×20–×40 magnification. 2: 4–10 dots/cell; very few dot clusters at ×20–×40 magnification. 3: >10 dots/cell; <10% have dot clusters at ×20 magnification. 4: >10 dots/cell; >10% positive cells have dot clusters at ×20 magnification. Score 0 was counted as a negative result and scores 1–4 as positive.

### Statistical analysis

All experimental data are expressed as mean ± SD of at least three independent experiments performed in triplicate, unless otherwise stated. Data are expressed as mean ± standard error (unless stated). A *p*-value of <0.05 was considered to be statistically significant.

### Ethical considerations

The study was approved after ethical reviews: 09/H1308/66 (generation of NOFs) and 13/NS/0120 (generation of CAFs) and 12/Lo/2018 (patient cohort). All the clinical cases were pseudo-anonymised before analysis.

## Results

### HPV status is linked to overall survival, but αSMA expression is only linked to overall survival in HPV-positive cases

**Figure 1:**
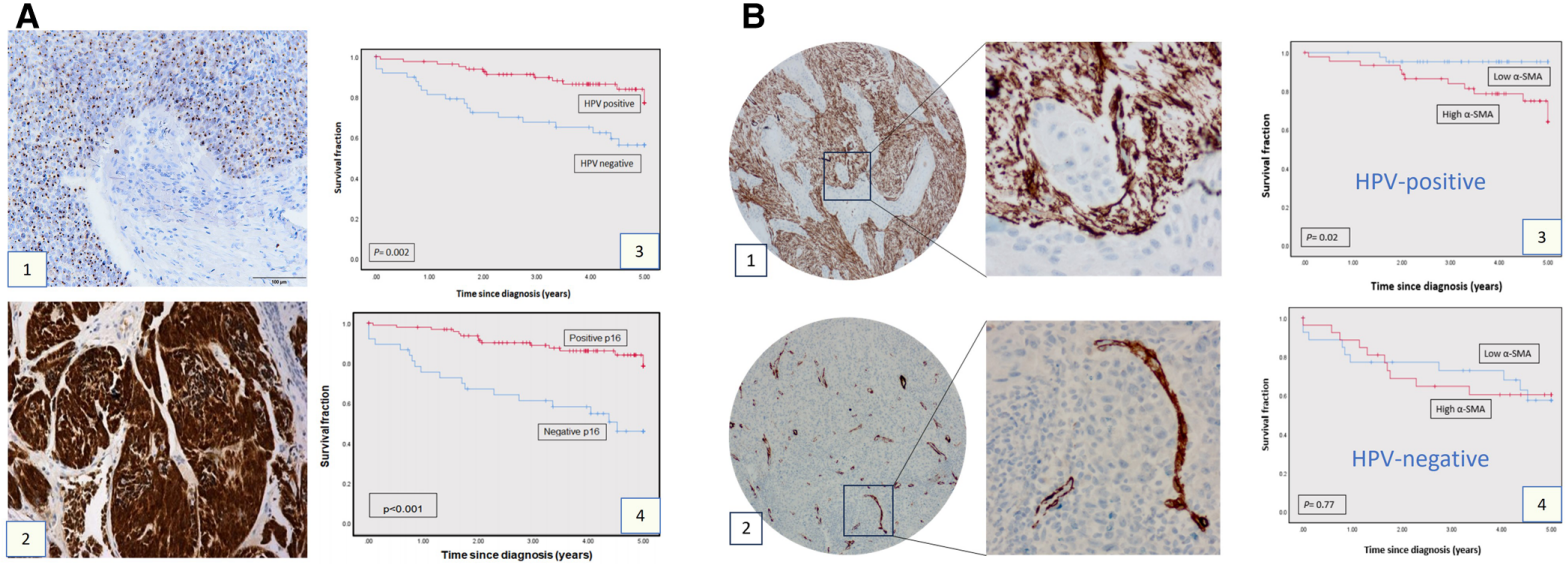
Clinical cohort TMAs: p16, HPV, SMA expression and survival. (**A**) p16 expression and HPV status in OPSCC cohort. HPV status as determined by HPV16/18 RNA ISH (1) and by p16 IHC (2). The survival curves at 5-years follow-up present overall survival using Kaplan-Meier method (Panels A3 and A4). The red curve represents the HPV or p16-positive group, and the blue curve represents the HPV-negative or p16-negative group. Each drop-step in the curve illustrates a case of death. In each the vertical line on the curve illustrates the last time seen for a live case. The HPV-positive and p16-positive groups demonstrated better overall survival (log rank 0.002 and 0.001 respectively). (**B**) α-SMA IHC expression in OPSCC in the OPSCC cohort. Anti ɑ-SMA monoclonal antibody (1:100) was used for IHC staining, showing high (1) and low (2) α-SMA expression. Images presented at 20X & 40X magnifications. Kaplan-Meier analysis for ɑ-SMA expression in the HPV-positive (3; *n* = 85: log rank 0.02) and HPV-negative group (4; *n* = 52, log rank 0.77). High ɑ-SMA expression is related to poorer overall survival in the HPV-positive OPSCC group. In each, the blue curve represents the ɑ-SMA-low group, and the red curve represents the ɑ-SMA-high group. Each drop-step in the curve illustrates a case of death. Each vertical line on the curve illustrates the last time seen for a live case.

The demographic details of the clinical cohort are presented in Table 1. Assessment of HPV status in the clinical cohort (*n* = 143) by both p16 IHC and HPV 16/18 RNA ISH demonstrated poorer overall survival related to the expression/presence of either biomarker (Figure 1A: p16 *p = 0.0002*, HPV ISH *p < 0.001*). In the assessment of *α*SMA expression (assessed as low or high: see Figures 1B1,B2), high *α*SMA expression was related to survival in only in HPV-positive cases (Figure 1B: HPV+ *p* = 0.02, HPV− *p = 0.77*).

### Normal tonsil fibroblasts respond to treatment with TGFβ and H_2_O_2_ in a very similar manner that that previously described in oral normal fibroblasts

**Figure 2:**
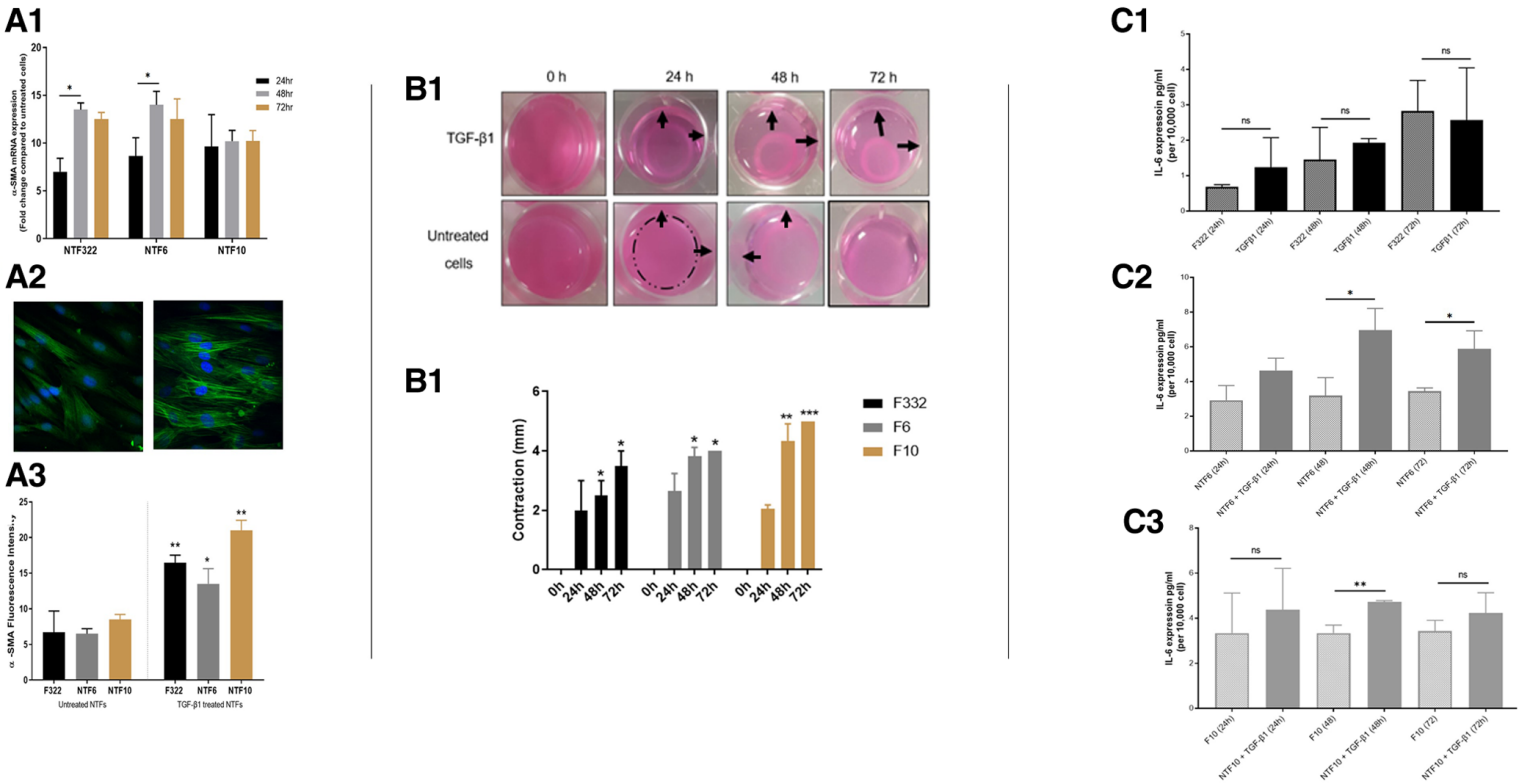
Characterization of NTF cultures in relation to TGFβ treatment. (**A**) Expression of SMA and formation of stress fibres in NFT cultures. Three NTF cultures were treated with 5 ng/ml TGFβ for 24, 48 and 72 h, and the induction of SMA as assessed by qPCR expression relative to B2M (A1), western blot with B-actin as loading control (A2) and immunofluorescence (A3 and A4, at 48 h): All showed response to TGFβ treatment, variably demonstrated in these assays, showing an increase in expression, maximal at 48 h, and all showing an increase in the development of SMA stress fibres. Data in bar charts is mean ±SEM. *p < 0.05, **p < 0.01. (**B**) Contraction of collagen matrix on treatment with TGFβ. B1: representative pictures of contraction of collagen matrix gels with NFT10 on treatment with 5 ng/ml TGFβ for 24, 48 or 72 h. B2: Image J quantification of contraction for all 3 cultures over the time course. Data presented is mean ±SD of three repeats *p < 0.05, **p < 0.01 ***p < 0.005. (**C**) production of IL-6 on TGFβ treatment of NTFs. Treatment with 5 ng/ml TGFβ over 24, 48 and 72 h resulted in increased production of IL-6 in NTF6 and NTF10, as measured by ELISA: IL-6 increased in NTF322 without TGFβ treatment. C1: NTF 322, C2: NFT6, C3 NTF10. Data presented is mean ±SEM of three repeats *p < 0.05, **p < 0.01.

We previously published a comprehensive characterisation of normal oral fibroblasts (NOF) in a overall similar experimental protocol (19), but it is possible that anatomical variations in mesenchyme may result in differences in fibroblast reactivity. Thus, we undertook characterisation of 3 new normal tonsil fibroblast (NTF322, NTF6 and NTF10) cultures, which allowed comparison with NOF cultures. We have demonstrated that all the NTF cultures respond to treatment with TGFβ1, with maximal effect seem at 48 h at transcript level (Figure 2A1), and 48–72 h at protein level (Figure 2A2). The development of αSMA stress fibres mirrored these changes (Figure 2A3). The functional consequences of this are seem in the increase in collagen gel contraction under the same treatment and timescale (Figure 2B), and in an increase in the production of IL-6, at protein level, but this was variable between the cultures and timepoints, with no increase noted in NTF322 (Figure 2C).

**Figure 3:**
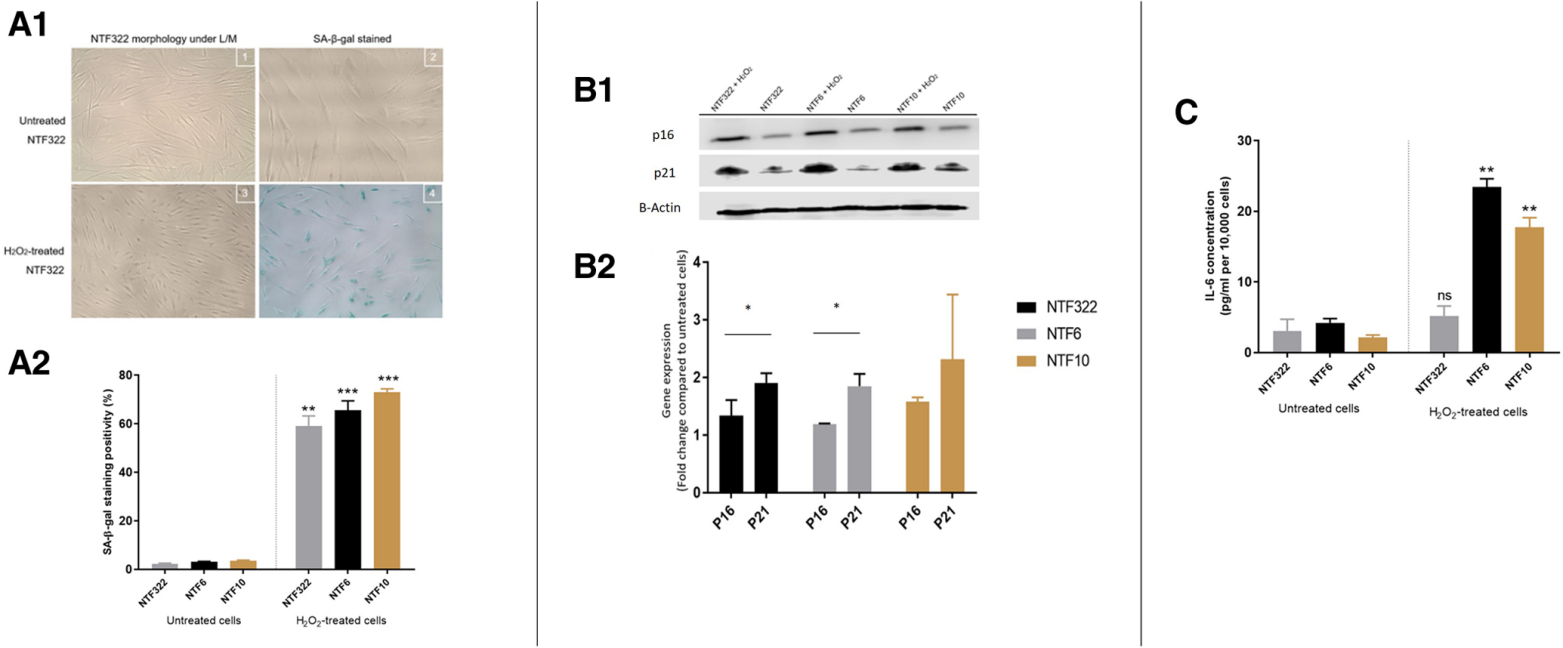
Characterization of NTF cultures in relation to H_2_O_2_ treatment for the induction of senescence. (**A**) Induction of senescence by H_2_O_2_ treatment in NTFs. Three NFT cultures were treated with 500 mM H_2_O_2_ for 2 h and maintained for 14 days to induce senescence. D1: Images representing SA-β-gal stain at basal level and after H_2_O_2_ treatment in NTF322. D2: Quantification of SA-β-gal stain. Data shows the mean proportion of cells stained blue in 3 random fields (magnification 20x). Confluent NTF6 were used as a positive control (not shown). Error bars = SEM. **p < 0.01, ***p < 0.001. (**B**) expression of cell cycle markers of senescence in NFTs. Western blot analysis of p21 and p16. NTF322, NTF6 and NTF10 were treated with 500 mM H2O2 for 2 h then maintained for 14 days. Protein expression of 20 μg of whole cell lysate per lane using anti-p21 antibody (1:1,000, R&D Systems, Minneapolis, US) or anti-p16 antibody (1:1,000, Abcam, Cambridge, UK), with B-actin as loading control. H_2_O_2_ treated NTFs showed increased levels of p16 and p21 at 14 days. E1: representative WB gel images. E2: Densitometry of Wester Blots showing mean of 3 repeats and SEM. *p < 0.05. (**C**) production of IL-6 by H_2_O_2_ treated NTFs. NTF322, NTF6 and NTF10 were treated with 500 mM H2O2 for 2 h, then maintained for 14 days. The media was collected and subjected to IL-6 ELISA analysis with untreated cells were included for comparison. All treated NTFs showed an increase in IL-6 production (*n* = 6). Y-axis denotes standardised IL-6 concentration in Pg per ml. IL-6 concentrations were optimised according to fibroblast density at 10,000 cells. **p < 0.01, ***p < 0.001. Data is mean ±SEM for 3 repeats.

Treatment with H_2_O_2_ resulted in a significant increase in the percentage of SA-β-Galactosidase activity (widely used as an indicator of senescence) in all cultures (Figure 3A), with associated increases in the expression of p16 and p21 in NTF332 and NTF6, assessed by western blot (Figure 3B). H_2_O_2_ treatment also resulted in an increase in the production of IL-6 in NTF6 and NTF10 (Figure 3C).

These results are very similar to those previously demonstrated using NOFs and a similar treatment schedule, with the induction of aSMA stress fibres on treatment with TGFβ1, and the induction of SA-β-Galactosidase activity on treatment with H_2_O_2_ (16).

### Characterisation of tonsil CAF cultures demonstrates differential baseline levels of senescence and reactivity to TGFβ

**Figure 4:**
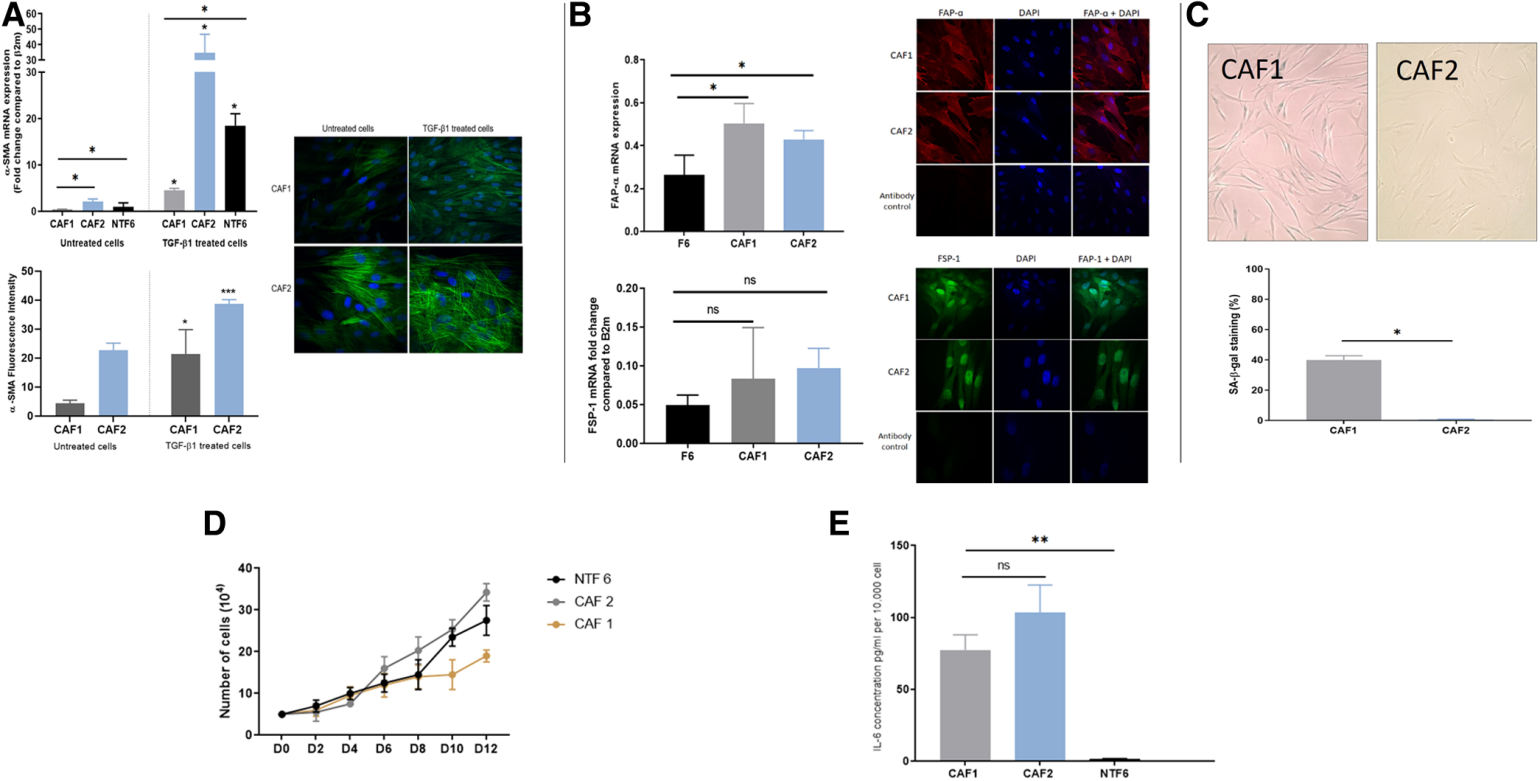
OPCAF characterization. (**A**) α-SMA in CAFs. Upper: α-SMA mRNA expression in OPSCC CAFs following TGFβ1 treatment. CAFs and NTF6 (250,000 cells/ well) were seeded in 6 well plates, starved for serum (24 h), then treated with TGFβ1 (5 ng/ml) for 24 h. Assessment of basal αSMA mRNA (untreated cells) revealed expression in CAF2 when compared to CAF1 and NFT6A. On treatment, significant upregulation in αSMA mRNA was seen in all tested cells (*p < 0.05) with the most striking increase in by CAF2. Error bars = SEM (*N* = 3, *n* = 3). Lower panels: Immunofluorescent representative photomicrographs demonstrated α-SMA basal and induced expression in OPSCC CAFs. Cell induction was performed by TGF-β1 (5 ng/ml) incubation (48 h). CAF2 displayed evident basal α-SMA expression. Images were taken using a Zeiss 880 Airy Scan confocal microscope, Magnification 40X. Quantification for detected ɑ-SMA fibres by image analysis was performed using Fiji-ImageJ. Error bar = SD (*N* = 3, *n* = 3). (**B**) FAP-α and FSP1 expression in CAFs. Upper: FAP-α expression in OPSCC CAFs and NTF6. (**A**), FAP-α mRNA expression in CAF1, CAF2, and NTF6. Error bars = SD (*N* = 2, *n* = 3). CAF1 and CA2 showed significant FAP-α expression compared to NTF6 (*p < 0.05). Student's *t-*test and one-way ANOVA were used for the statistical comparison. Immunofluorescence staining of FAP-α, all tested cells exhibited evident homogenous FAP-α protein using (1:100) anti-FAP-α monoclonal antibody. Images were taken using a confocal microscope (Zeiss 880 airy Scan). Magnification (x40). Lower: FSP-1 mRNA expression in CAFs and NTF6. CAF1 and CAF2 showed higher FSP-1 expression compared to NTF6. Error bars = SEM (*N* = 3, *n* = 3). Immunofluorescence staining of FSP-1, All tested cells exhibit evident homogenous FAP-α protein cytoplasmic and nuclear expression using (1:100) anti-FSP-1 monoclonal antibody. Images were taken using a Zeiss 880 Airy Scan confocal microscope, magnification (x40). (**C**) Senescent cells in proliferating CAF1 and CAF2 cultures. Upper: Images representing SA-β-gal stain in CAFs at the basal level (magnification x20). Cells were seeded into a 12 well plate at a density of 10,000 cells/well. (**B**) Quantification of SA-β-Gal stain. Mean number of stained cells (blue precipitate) in 3 random fields (magnification x20). CAF1 showed the highest percentage of senescence. Error bar = SEM of (*N* = 3, *n* = 3). *p < 0.05. (**D**) Growth curves of CAF1 and CAF2 with NTF6 for comparison. 5,000 cells of CAF1, CAF2, and NTF6 were plated into 6-well plates. Cell counting was performed at 2-day intervals. CAF1 showed the slowest proliferation rate. Each line on the figure represents the mean of relative cell number. Error bar = SEM of three independent experiments. (**E**) Background IL-6 production in proliferating cultures of CAF1, CAF2 and NTF6 by ELISA. Y-axis denotes IL-6 concentration in pg/ml, normalised to fibroblast density per 10,000 cells. Student's *t*-test and one-way ANOVA were used for the statistical comparison. CAF1 and CAF2 showed significantly higher IL-6 concentration than NTF6. Data represent the mean of (*N* = 3, *n* = 3). Error bars = SD. **p < 0.01.

A similar process of characterisation for the two new tonsil CAF cultures (CAF1 and CAF2) was undertaken, after confirmation of lack of contamination of these cultures by other cell types. qPCR assessment of cytokeratin 6, HLA-DR and CD31 demonstrated no significant expression in the cultures, indicating lack of epithelial, lymphocyte and endothelial cell contamination (Supplementary Figure S1). The two CAF cultures, which were both derived from HPV-positive tumours in patients who were both current smokers varied in their phenotype (Figure 4). Whilst both CAF1 and CAF2 increased their expression of *α*SMA (mRNA transcript and stress fibre formation; Figure 3A), the baseline expression of αSMA was higher in CAF2 than in CAF1. We also identified higher expression of FAP-α and FSP1 in the CAFs when compared to NTF6 (Figure 4B), and similar patterns of expression of PDGRA (data not shown). On assessment of the proportion of senescent cells at baseline, there was a higher proportion of senescent cells in CAF1 cultures (Figure 4C). Treatment of CAF1 and CAF2 with H_2_O_2_ resulted in an increase in the proportion of SA-β-Gal expressing cells, with the dynamic change greater in CAF2 (Supplementary Figure S2).

The assessment of growth curves, with NTF6 for comparison, also demonstrated differences between CAF1 and CAF2, with the rate of increase in cell numbers less in CAF1, likely due to the higher proportion of senescent cells in these cultures (Figure 4D). Notably, both CAF cultures produced a much higher baseline level of IL-6 production when compared to NTF6, in keeping with the higher baseline SA-β-Gal and αSMA expression (Figure 4E).

### Culture of tonsil fibroblasts with conditioned media from OPSCC cultures results in differential cytokine expression when comparing media from HPV-positive and HPV-negative cells

In our previous OPSCC paper, we demonstrated the baseline cytokine production of the OPSCC cells (19). The current assessment, which includes a small number of additional cytokines not assessed previously, was undertaken on untreated CAF1, CAF2 and NTF322 cultures (Supplementary Figure S3). This demonstrated a limited baseline repertoire of chemokine production: In CAF1 and CAF2, only CCL2 and MMP2 were detected (with weak expression of SPARC in CAF2), whilst SPARC, Serpin B5 and DCN were detected in NTF322 conditioned media.

**Figure 5:**
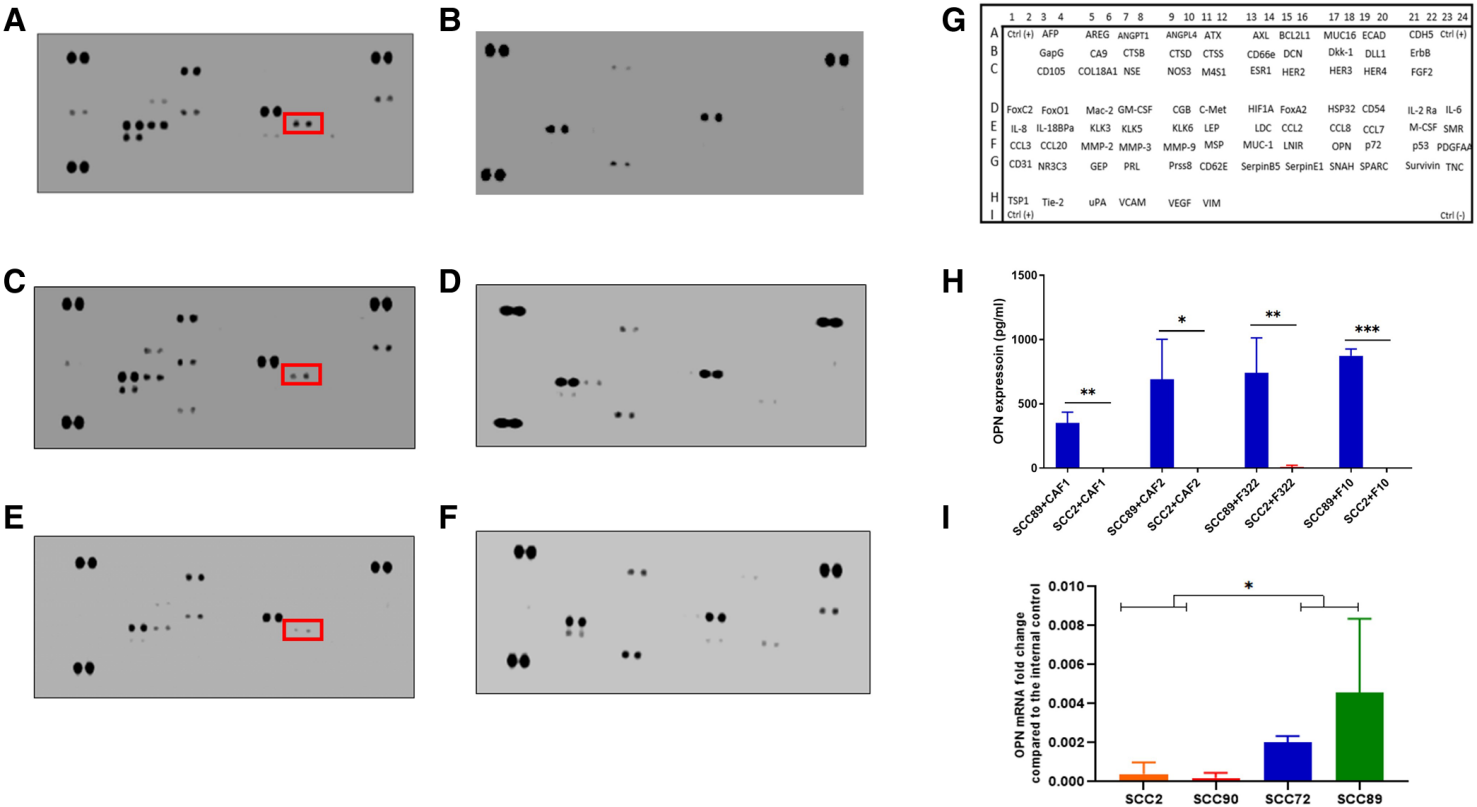
Cytokine array analysis of CAF conditioned media, following stimulation with OPSCC cell conditioned media for 24 h. Array images show the developed cytokine membrane for media collected after CAF treatment with OPSCC CM. The array images are (**A**) CAF1 stimulated by SCC89 CM (**B**) CAF1 stimulated by SCC2 CM, (**C**) CAF2 stimulated by SCC89 CM, (**D**) CAF2 stimulated by SCC2 CM, (**E**) NTF322 stimulated by SCC89 CM, (**F**) NTF322 stimulated by SCC2 CM. Panel (**G**) shows the cytokine array map. The detected spots are summarized in Supplementary Table S5. Osteopontin (SPP1) is highlighted in each of the cytokine arrays from HPV negative OPSCC CM. (**H**) validation of the array data by qPCR in HPV positive cells (SCC2 and SCC90; top panel) and by ELISA in the 24 h combined conditioned media (SCC2 and SCC89, with CAF1, CAF2 and NTF322 in turn; bottom panel). A significant difference between HPV negative and HPV positive cell lines is detected. Data represent the mean of (*N* = 3, *n* = 3). Error bars = SD. *p < 0.05, **p < 0.01, ***p < 0.005.

After 24 h of treatment of the fibroblast cultures with OPSCC CM, two different collections of conditioned media were made: firstly, the initial 24 h CM, which contains cytokines from both the SCC cells and fibroblasts, and after washing and incubation for a further 24 h in fresh media, the second CM was collected (fibroblast produced cytokines only). The initial conditioned media (CM1) was subjected to cytokine array analysis as shown in Figures 5A–F. The chemokines which were detected are presented in Supplementary Table S5. Several cytokines were detected in the CM which was originally derived from HPV+ and HPV− cells, some of which were also detected in the baseline fibroblast media (Supplementary Figure S3), including CCL2. Increased production was detected solely related to HPV+ cell CM. The cytokine profiles are much reduced in the later CM2 (Supplementary Figure S4), but with a notably increased repertoire still evident in fibroblast CM which has been stimulated by HPV− SCC CM, when compared to HPV+.

We identified OPN (SPP1) as a consistently produced cytokine present in the CM1 from all fibroblast cultures in response to SCC89 CM (HPV negative). Validation of the findings from the cytokine array by ELISA in CM1 demonstrated similar findings (Figure 5H), but OPN is not detected in CM2 (Supplementary Figure S4), which indicates that the source is not the fibroblast cells. qPCR assessment of the OPSCC cells indicated variable baseline transcription of SPP1, higher in HPV-negative cells (SCC 72, SCC89) than in HPV positive (SCC2, SCC90) cells (Figure 5J). We also validated the production of CCL2 in CM1 by ELISA, with no difference in SCC89 and SCC2 CCL2 production in response to CAF1 and CAF2 CM, but variable when stimulated by NTF media (data not shown).

### Transwell co-culture methods identify SCC cells as the primary source of osteopontin and fibroblasts as the source of IL-6

**Figure 6:**
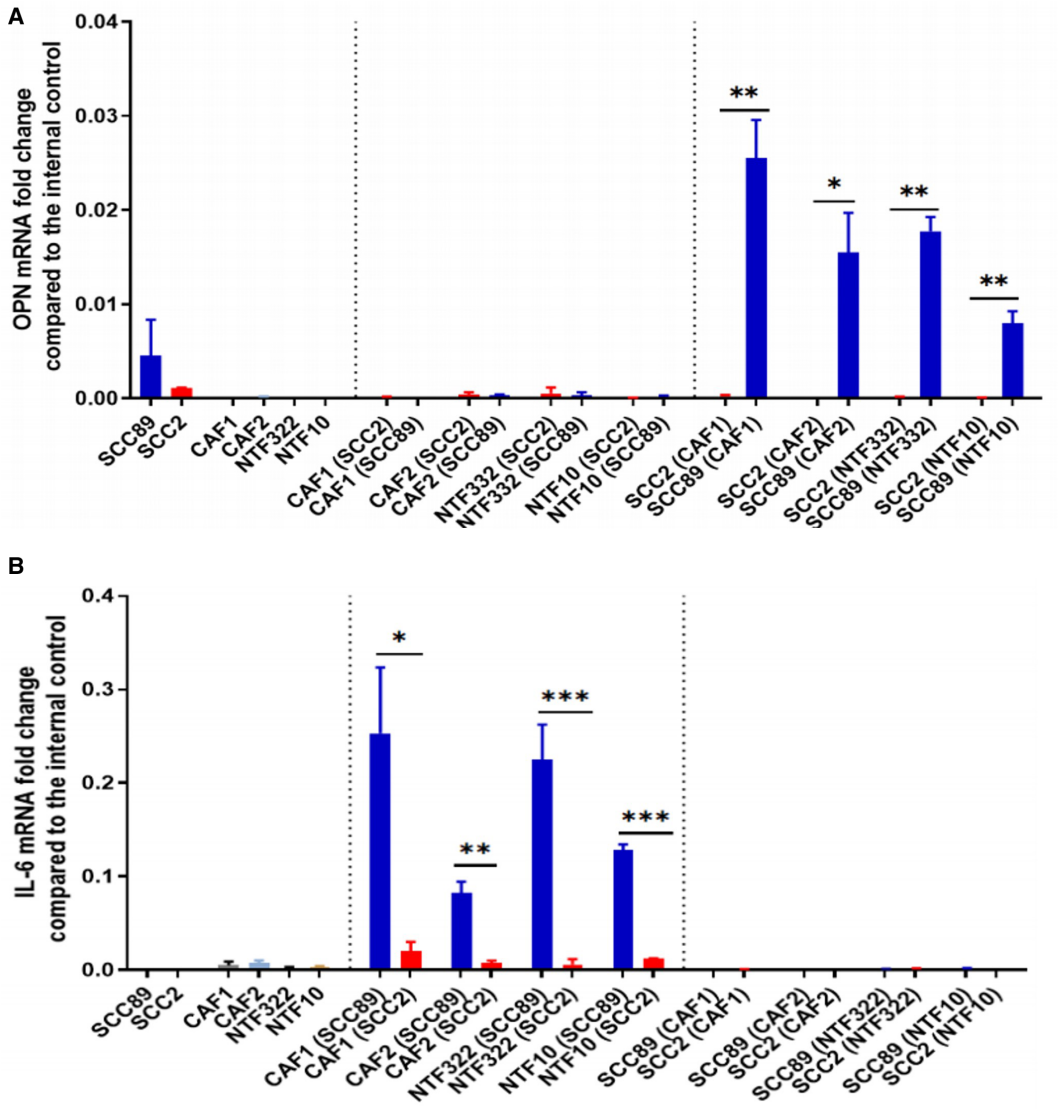
Co-culture models show fibroblasts release IL-6 which results in OPN release from OPSCC cells. SCC89 or SCC2 (3 × 10^5^ cells) were co-cultured with OPSCC fibroblasts (CAF1, CAF2, NTF322, NTF10) (50,000 cells) using a transwell insert (0.45 μm) for 48 h (SCC cells upper, Fibroblasts lower). The cell pellets were collected separately. OPN (**A**) and IL-6 (**B**) mRNA expression was analysed using qRT-PCR and untreated cells were included as a control. OPN and IL-6 fold change is relative to β2m expression. Blue bars denote HPV-negative SCC cultures (SCC89), and red bars denote HPV-positive cultures (SCC2). Data represent the mean of (*N* = 3, *n* = 3), except where indicated. (**A**) Transwell co-culture model demonstrates that OPN is produced by HPV- OPSCC cells, not HPV+ OPSCC or NTFs. Co-cultured SCC89s showed higher OPN mRNA expression compared to Co-cultured SCC2s, with little/no expression in fibroblast cultures. Error bars = SD. *p < 0.05, **p < 0.01. (**B**) Transwell co-culture model demonstrates that IL-6 is produced by fibroblasts, but only when cultured with HPV- OPSCC cells, not with HPV+ OPSCC cells. Co-cultured SCC89s showed higher IL-6 mRNA expression compared to Co-cultured SCC2s. Error bars = SD. Data represent the mean of (*N* = 3, *n* = 3), except in co-cultured cancer cells where (*N* = 2, *n* = 3). Statistical analysis was determined using two-tailed Student *t*-test with *p < 0.05 and **p < 0.01 ***p < 0.005.

We used a transwell co-culture method to allow the cells to communicate in a paracrine manner but with no direct contact, allowing for separate transcript analysis of the different cells used. Assessment of OPN transcript levels by qPCR indicated that these were only elevated in the HPV-negative cells when co-cultured with either CAFs or NTFs (Figure 6A). No expression was seen either in HPV-positive SCC cells or in CAFs or NTFs. Conversely, IL-6 transcript was increased only in CAFs and NTFs when cultured with conditioned media from HPV-negative SCCs (Figure 6B). no increase was seen in the OPSCC cells of in CAFs/NTFs cultured with HPV-positive SCC conditioned media.

### Treatment of tonsil fibroblasts with recombinant osteopontin results in increased contraction and IL-6 production which can be inhibited by receptor blocking antibodies

**Figure 7:**
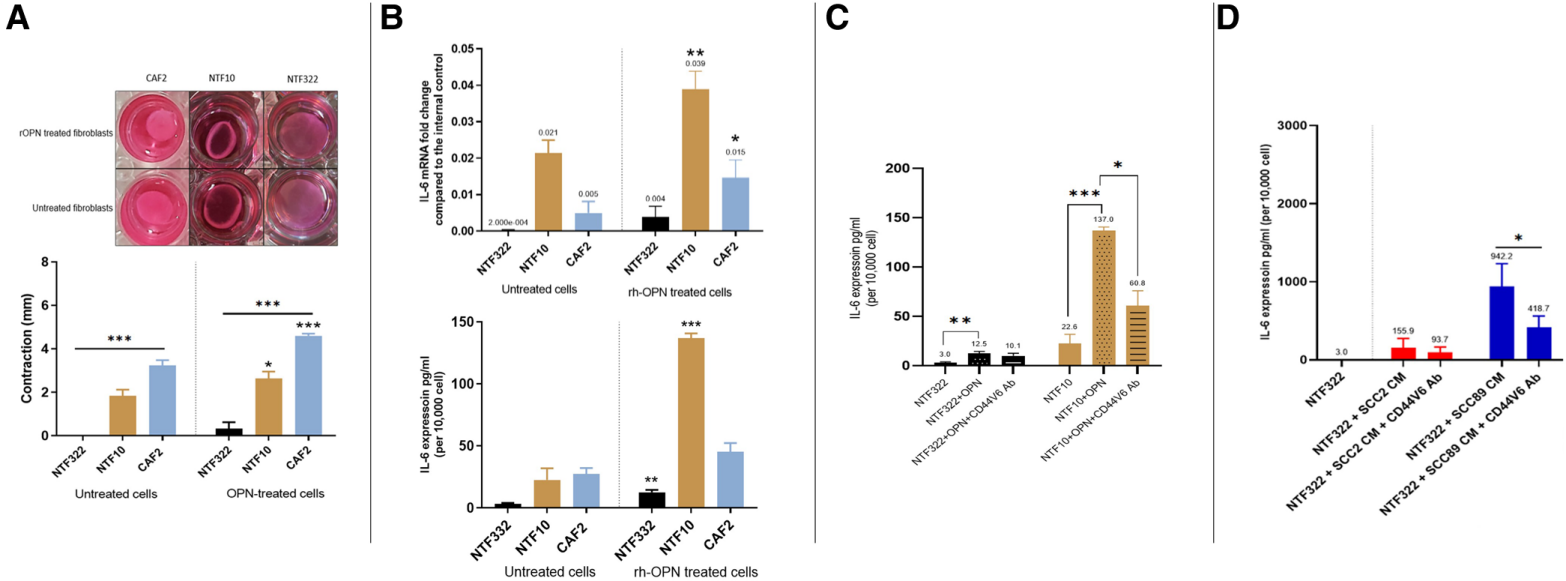
Effects of recombinant OPN on fibroblasts and OPSCC cells. (**A**) Matrix contraction ability of rOPN treated NTFs varies between different cultures Top panel: representative gel contraction images of 2 × 10^4^ cells seeded in 0.5 ml of collagen lattice. 1.0 ml of growth media was added on the top of the collagen gel lattice and the plate was incubated for 24 h. The next day, the cells were serum-starved for further 24 h, then were treated with 180 ng/ml OPN before releasing the stressed gel matrix from the walls of the well. The gels were then incubated for 48 h before measurement. Bottom panel: Quantification of collagen gel disc contraction using ImageJ. Relative lattice contraction (mm) was obtained by measuring the distance between the gel and the well border. NTF322 was much less responsive than NFT10 and CAF2. Data represent the mean of (*N* = 3, *n* = 3). Error bars = SEM. *p < 0.05, ***p < 0.001. (**B**) IL-6 upregulation following rOPN treatment. OPSCC Fibroblasts and NTFs were cultured to 70% confluence then treated with 180 ng/ml rOPN for 48 h. Top: The cell pellet was collected, and qRT-PCR was used to analyse IL-6 expression, normalised to β2m. Bottom: media was collected and IL-6 ELISA undertaken. NTF10 and CAF2 showed increased IL-6 transcript levels compared to the untreated counterparts (*p *< 0.01), and NTF322 and NTF10 showed increased IL-6 secretion. Error bars = SD for (*N* = 3, *n* = 3). *p < 0.05, **p < 0.01, ***p < 0.001. (**C**) Anti CD44v6 antibody reduced IL-6 production in NTFs when treated with rOPN. NTF322 and NTF10 were cultured to 70% confluence in T75, then incubated with 5 μl/ml of anti-CD44v6 antibody for 1 h, followed by incubation with 180 ng/ml of rOPN for 24 h. Controls were incubated with serum-free media only or rOPN only without blocking antibody. The media was collected and subjected to IL-6 ELISA analysis. IL-6 concentrations were optimised according to a fibroblast density of 10,000 cells. Error bars = SD for (*N* = 3, *n* = 3). *p < 0.05. **p < 0.01 and p < 0.001. (**D**) Anti CD44v6 antibody treatment reduces the production of IL-6 in fibroblasts on OPSCC CM treatment. NTF322 and NTF10 were cultured to 70% confluence, then incubated with 5 μl/ml of anti-CD44v6 antibody for 1 h, followed by incubation with SCC2, SCC89 or SCC72 CM for 24 h. Untreated counterparts were incubated with serum-free media or CM only without blocking antibody. The media was collected and subjected to IL-6 ELISA analysis. IL-6 concentrations were optimised according to a fibroblast density of 10,000 cells. Error bars = SD for (*N* = 3, *n* = 3). *p < 0.05.

Having established that OPN was being produced by the HPV-negative SCC cells in response to fibroblasts, we treated CAF2 and NTF cultures with recombinant OPN (rOPN). This resulted in increased contraction of collagen gels for NTF10 and CAF2, with increased production of IL-6 (transcript and protein) also detected (Figures 7A,B). The increase in IL-6 production could be reduced, but not abolished, by pre-treatment with anti-CD44V6 antibody (Figure 7C), in keeping with data which demonstrates OPN binding to proteins other than its specific receptor, such as multiple integrins (22). Pre-treatment of fibroblasts with anti-CD44V6 before culture with OPSCC conditioned media showed a reduction in IL-6 production in fibroblasts which were treated with HPV-negative SCC CM, whilst no significant difference was noted in fibroblasts which had been cultured in HPV-positive SCC CM (Figure 7D).

## Discussion

The role of fibroblasts in the tumour microenvironment of OPSCC has been understudied, even when compared to those at other sites within the head and neck. Others have shown that CAFs are frequently identified in the TME of HPV+ OPSCC (23), but, in general, the data (IHC and sequencing) suggests higher fibroblast signatures in HPV-negative OPSCC when compared with HPV-positive OPSCC (18). The data which we have generated from the assessment of the OPSCC TMAs would bear this out (Figure 1). Other investigators have demonstrated that stromal desmoplasia (by morphological assessment alone or by *α*SMA expression) is associated with poorer disease specific survival in both HPV+ and HPV-negative (24), whereas we only found this relationship in HPV-positive tumours (Figure 1).

Expression of various CAF markers in OPSCC has been investigated by others who have reported the involvement of members of the Fibroblast Growth Factor Receptor family: FGFR1 has been reported as more highly expressed in HPV-negative OPSCC, but not related to outcome (25): FGFR3 expression correlates with mutant p53 expression and poor survival in p16-negative OPSCC (26): FGFR4 expression has been described in OSCC and OPSCC, without any link to survival (27). Other stromal fibroblast associated markers, such as PDGFR-a, may be activating mesenchymal stem cell in the HNSCC TME to form CAFs (28). In the limited studies available, most demonstrate both qualitative and quantitative differences between the TME of HPV-positive and HPV-negative tumours.

In this study, we have expanded on our earlier work characterising the interaction between normal oral fibroblasts and OPSCC cells (19). The earlier investigations highlighted the importance of IL-6 in cytokine-mediated communication between NOFs and OPSCC cells, and our initial aims were to determine if tonsil-derived fibroblasts, both normal (Figures 2, 3) and cancer-derived (Figure 4) would behave in a similar manner. We have now demonstrated that NOFs and NTFs respond similarly to each other in terms of phenotypic changes on stimulation with TGF-β1 and H_2_O_2_ [Figure 2, compare with (19]), but also in their interactions with OPSCC cells (Figure 4). Similar features have been demonstrated in the interaction of cervical cancer cells and cervix CAFs in culture (29).

In this study, we also report the characterisation of two CAF cultures derived from OPSCCs (Figure 4). These fibroblast cultures vary in their basal expression of αSMA and SA-β-Gal activity (as a surrogate marker of senescence), but both can interact with the HPV-negative OPSCC cells in a similar manner (Figure 5). This, however, does raise an interesting issue: that CAFs vary in phenotype is now very evident from single cell sequencing analyses (14, 30) and that these are related to various roles in the TME, including immune infiltration. However, the question of whether all the described CAF phenotypes result in distinct functional states still requires to be addressed in detail. In this context, we have demonstrated that OPN is another partner in IL-6 driven communication between cells in the TME, which impinges on many of these varied CAF functions. A further point to note is the age difference between the two patients from whom the CAFs were derived. This may have resulted in differences in senescence due to aging and not to the carcinogenic process, and it is difficult to draw wider conclusions form only 2 CAF cultures. This will require to be taken into account when assessing the effects of senescence across a larger patient population.

This investigation confirms our earlier observation of the importance of IL-6/STAT signalling in the communication between cells in the OPSCC TME (19), an effect which is limited to HPV-negative tumour cells in culture. The role of the IL-6 pathway has been described in many cancers, linked to formation of an inflammatory TME, in addition to effects in the tumour cells themselves via the JAK/STAT signalling pathway (31). In HPV-negative OPSCC, IL-6 has been associated with poor prognosis, but this relationship has not been noted in HPV-positive OPSCC (32). We have previously demonstrated that the effects of IL-6 from fibroblasts may be, in part mediated via the production of HGF by HPV-negative OPSCC cells (19). Whereas other have associated this with nuclear localisation of PRMT5 or the expression of DEK expression (33). Similar findings have been demonstrated in cervical cancer, where IL-6/STAT3 signalling activates fibroblasts and increases senescence (in CAFs, but not normal fibroblasts) (34), increases in αSMA expression (35), and wider effects on other aspects of the TME include TH17 cells (which may contribute to immunosuppression in the TME (36).

Our investigations, using CAFs and a wider panel of cancer-associated cytokines, has identified osteopontin (OPN/SPP1) as a further mediator of the effects of CAF/OPSCC crosstalk, but again, only in HPV-negative cells (Figures 5, 6). The pattern of CCL2 production was not consistent in this system, and thus was not pursed further. In our co-culture investigation, we have demonstrated that the source of OPN in this system is the HPV-negative OPSCC cells and that OPN, in turn, acts on the fibroblasts to increase IL-6 production and increase contraction of collagen gels (Figures 7A,B). These effects can, in part, be abrogated using a CD44v6 inhibiting antibody (Figures 7C,D), demonstrating the OPN is only one of several mediators which may be acting in this manner (including HGF).

OPN is well recognised as a key mediator in the in TME of many malignant tumours, largely studied in the context of tumour associated macrophages (TAMs). Increased expression has been associated with reduced survival in a variety of tumours, with correlation with the extent of immune infiltration (37). Descriptive and mechanistic studies have also demonstrated a link with chemoresistance in ovarian (38), hepatocellular (39) and lung (40) malignancies. CAFs may interact with SPP1-expressing macrophages in the TME, and this feature has been associated with the EMT-like CAF phenotype (41). In colorectal carcinoma, a combination of FAP-expressing fibroblasts and OPN-expressing TAMs corelate with poor responses to immune checkpoint (42), and indeed, in poorly differentiated tumours, OPN in TAMs has been shown to induce senescence in tumour cells in high grade tumours, with the development of a profound SASP which may drive tumour progression (43).

In 2003, a paper was published identifying SPP1/OPN as a plasma prognostic marker in HNSCC, but few studies have followed up on the biology of this observation (44). SPP1 expression has subsequently been associated with poor outcomes in HNSCC (without subsite analysis) and with resistance to cetuximab (45, 46). It appears likely that the activity of OPN in HNSCC may be paracrine or autocrine in manner, and that this includes OPN-expressing macrophages, which have not been studied in this paper. SPP1-expressing TAM subpopulations have been shown to promote intravasation and metastasis in HNSCC (47) and low TAM containing tumours (CD68 CD163) have been associated with better survival (48).

Interestingly, given the lack of response between the CAFs and HPV-positive cells which we have again demonstrated, many more M1/M2 macrophages (which often express OPN) have been identified in HPV+ stroma, which, if at a high level, has been associated with poor regional control (49). It has also been demonstrated that macrophages expressing TREM1 are important in the progression of HPV+ OPSCC (50) and that fibroblasts with a matrix-CAF phenotype collaborate with SPP1-expressing TAMs to promote tumour progression in HNSCC (51). This may indicate a greater importance in the role of TAMs in the HPV-positive TME, which will require further investigation, as an additional element to the OPSCC-fibroblast model.

## Conclusion

Whilst our investigations of the interactions between OPSCC and oropharynx fibroblasts have identified the role of OPN, these observations further serve to underline the complexity of intercellular communication in the OPSCC TME. We have demonstrated that OPN release from HPV-negative OPSCC cells was stimulated by IL-6 from OP CAFs. OPN resulted in contraction of OP CAFs and further release of IL-6. As in our previous investigations, the HPV-positive cells were much less responsive to fibroblast conditioned media in terms of their secretome and addressing the issues this raises regarding the role of the TME in HPV-positive tumours may only be possible using comparative single-cell sequencing of HPV positive and HPV negative tumours.

## Data Availability

The raw data supporting the conclusions of this article will be made available by the authors, without undue reservation.

## Supplementary material

Supplementary Figure S1 Demonstration of lack of CK6, HLA-DR and CD31 expression in cultures in NTFs and CAFs. (A) Cytokeratin 6 expression: CAF1 and CAF2 cells were collected, and total RNA extracted, and qRT-PCR was performed to amplify CK6 with β2m used for normalisation. NOK8 used as positive control and NTF6 as negative control. CAFs culture exhibited negligible expression of CK6 suggesting cultural purity. ****p* < 0.000, using one-way ANOVA. Error bars = SD of (*N* = 3, *n* = 3). (B) HLA-DR expression: OPSCC CAFs clear of immune cell contamination. CAF1 and CAF2 were collected, and total RNA was extracted and quantified. qRT-PCR was performed to amplify the HLA-DR gene with β2m used for normalisation. MDM cells were used as positive control. CAFs culture exhibited a negligible expression of HLA-DR. ****p* < 0.001, using one-way ANOVA. Error bars = SD of (*N* = 2, *n* = 3). (C) CD31 expression OPSCC CAFs cultures are clear of endothelial cell contamination. CAF1 and CAF2 were collected, and total RNA was extracted and quantified. qRT-PCR was performed to amplify the CD31 gene with β2m used for normalisation. HDMEC were used as positive control. CAFs culture exhibited a negligible expression of HDMEC. ****p* < 0.001, using one-way ANOVA. Error bars = SD of (*N* = 2, *n* = 3).

Supplementary Figure S2 Induction of senescence by H2O2 treatment in OPCAFs and NTF6. The three cultures were treated with 500mM H2O2 for 2h and maintained for 14 days to induce senescence. Quantification of the % of cells stained for SA-β-gal: data shows the mean proportion of cells stained blue in 3 random fields (magnification 20x). Confluent NTF6 were used as a positive control. **p* < 0.05, ***p* < 0.01, ****p* < 0.005. Error bars = SEM.

Supplementary Figure S3 Baseline CAF1, CAF2 and NFT322 cytokine production. Array images show the developed cytokine membrane for CAF media without treatment. The array images are (A) CAF1 CM: Only CCL2 and MMP2 were detected. (B) CAF2 CM: MMP2, CCL2 and SPARC (weak) were detected. (C) NTF322 CM: MMP2 spots showed strong expression, other cytokines (SPARC, Serpin B5, DCN) showed weak signals. The cytokine array map is also included (D).

Supplementary Figure S4 Cytokine array analysis of CAF conditioned media, collected after application of fresh media, after the treatment of the fibroblasts with OPSCC CM for 24h. The array images are (A) CAF1 stimulated by SCC89 CM (B) CAF1 stimulated by SCC2 CM, (C) CAF2 stimulated by SCC89 CM, (D) CAF2 stimulated by SCC2 CM, (E) NTF322 stimulated by SCC89 CM, (F) NTF322 stimulated by SCC2 CM. The number of cytokines identified is reduced when compared to the combined CM presented in Figure 4, but represents the cytokines produced by the fibroblasts alone. Of note, a greater range of cytokines is present in the media from cells which have been treated by HPV-negative OPSCC CM (A, C and E). Of note, osteopontin (SPP1) is not identified, suggesting that it is produced by the SCC cells.

Supplementary Figure S5 Quantification of the proportion of senescent cells after rOPN treatment of NTFs. (A) Images representing SA-β-gal stain at basal level and after OPN treatment in NTF322 and NTF10, 10X magnification. (B) Quantification of SA-β-gal stain. The proportion of stained cells (blue precipitate) in 3 random fields at magnification 20x. Statistical analysis was performed using Student's *t-test*. Error bars = SD for (*N* = 3, *n* = 3).
