# Supplementary material for: Fibroblasts from HPV-negative oropharynx squamous cell carcinomas stimulate the release of osteopontin from cancer cells via the release of IL-6
Source: Front Oral Health. 2024 May 13;5:1390081. doi: 10.3389/froh.2024.1390081 (PMC11128591; doi:10.3389/froh.2024.1390081)
Supplement: Supplementary file 1 [file Table1.docx]

**Supplementary tables**

Table S1: Summary of epithelial cells lines

Table S2: Summary of fibroblast cultures

Table S3: qPCR primers. The SSP1 TaqMan probe covers the five isoforms of secretory-type OPN

Table S4: Primary antibodies used in western blot and immunofluorescence.

Table S5: Cytokine array differential expression list

| Cell line | Clinical details | Site | Age | Sex | Smoker? | Alcohol? | HPV status |
| --- | --- | --- | --- | --- | --- | --- | --- |
| SCC89 | New primary | Tonsil | 58 | M | Y | Y | Negative |
| SCC72 | New primary | Tonsil | 61 | F | Y | Y | Negative |
| SCC2 | NK | Hypopharynx | NK | M | Y | Y | Positive |
| SCC90 | Recurrence | Base of tongue | 46 | M | Y | Y | Positive |

**Table S1: Summary of epithelial cell lines.** NK= not known.

| Cell culture | Sex | Age | Site | TNM7 | Tumour HPV status |
| --- | --- | --- | --- | --- | --- |
| NTF6 | F | 29 | Tonsil | - | - |
| NTF10 | F | 19 | Tonsil | - | - |
| NTF322 | F | 21 | Tonsil | - | - |
| CAF1 | M | 61 | Tonsil | T2 N1 | Positive |
| CAF2 | M | 80 | Tonsil | T2 N3b | Positive |

**Table S2: Summary of fibroblast cultures**

| **TaqMan probes** | |
| --- | --- |
| Name | HS number |
| HLA-DR | 00219575 |
| CD31 | 01065279 |
| FAP-α | 00159849 |
| **PDGFRα** | 00183486 |
| IL6 | 00985639 |
| CDKN1a | 00355782 |
| CDKN2a | 00923894 |
| SPP1 (OPN) | 00960942 |
| β2M | 4325797 |
| **SYBR Green** | |
| αSMA | F: 5’ GAAGAAGAGGACAGCACTG 3’  R: 5’ TCCCATTCCCACCATCAC 3’ |
| FSP1 | F: 5’ TGTAATTGTGTCCACCTTCG 3’  R: 5’ GCTCATCACCTTCTGGAATG 3’ |
| CK6 | F: 5’ GTCCTCAGGCCCCTCTCTGG 3’  R: 5’ CCCCTGGCAATTTTCTGCAA 3’ |

**Table S3. qPCR primers. The SSP1 TaqMan probe covers the five isoforms of secretory type OPN**

| Primary antibody | concentration | Source |
| --- | --- | --- |
| αSMA | 1:1000 (WB)/1:100 IF | Sigma Aldrich |
| p16 | 1:1000 | Abcam |
| p21 | 1:1000 | R&D systems |
| OPN/SPP1 | 1:1000 | Proteintech |
| β-Actin | 1:3000 | Sigma Aldrich |

**Table S4. Primary antibodies used in western blot and immunofluorescence.**

|  | SCC 89 and SCC2 | SCC89 only | SCC2 only |
| --- | --- | --- | --- |
| CAF1 | Cathepsin D  IL6  CCL2  **MMP3**  **Serpin E1** | **Decorin**  **IL8**  GM-CSF  OPN  **Progranulin**  KLK6  Serpin B5  SPARC | VEGF |
| CAF2 | Cathepsin D  IL6  IL8  **CCL2**  Progranulin  **MMP3**  VEGF | GM-CSF  KLK6  OPN  EpCAM  C-MET |  |
| NTF322 | Cathepsin D  IL6  IL8  CCL2  Progranulin  VEGF | GM-CSF  MMP3  OPN  KLK6 | DKK-1  Lumiacan |

**Table S5:** Summary of differentially expressed cytokines produced in initial 24h media (M1): CAF1, CAF2 and NTF322 incubated with SCC89 or SCC2 conditioned media. Those highlighted in bold are also present in fibroblast-only (M2, post 24h) media. Additionally, IL6 and IL8 are present in M2 media, but only in SCC89 CM stimulated fibroblasts.
