# Supplementary figures and images for: Fibroblasts from HPV-negative oropharynx squamous cell carcinomas stimulate the release of osteopontin from cancer cells via the release of IL-6
Source: Front Oral Health. 2024 May 13;5:1390081. doi: 10.3389/froh.2024.1390081 (PMC11128591; doi:10.3389/froh.2024.1390081)

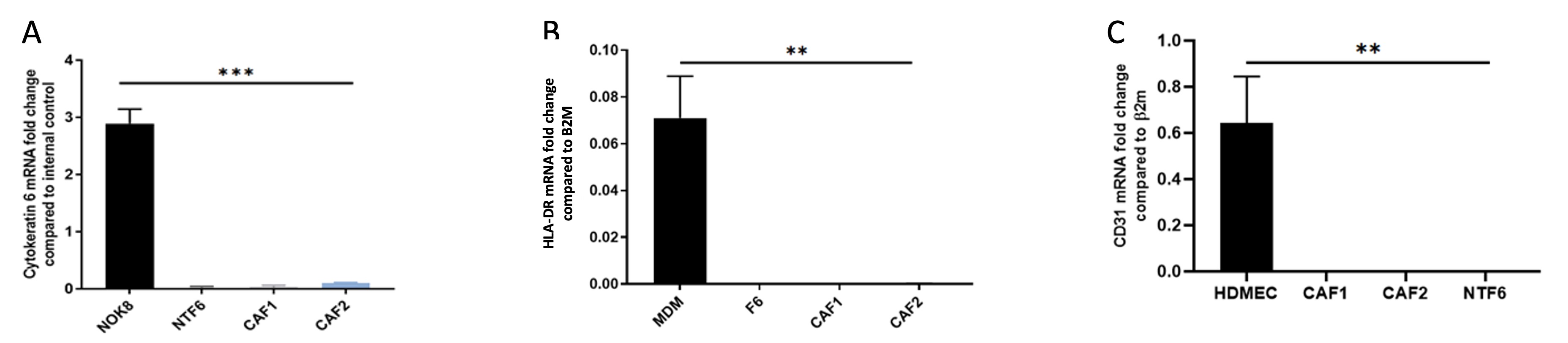

Supplement: Supplementary Figure S1 — Demonstration of lack of CK6, HLA-DR and CD31 expression in cultures in NTFs and CAFs. (A) Cytokeratin 6 expression: CAF1 and CAF2 cells were collected, and total RNA extracted, and qRT-PCR was performed to amplify CK6 with β2m used for normalisation. NOK8 used as positive control and NTF6 as negative control. CAFs culture exhibited negligible expression of CK6 suggesting cultural purity. ***p < 0.000, using one-way ANOVA. Error bars = SD of (N = 3, n = 3). (B) HLA-DR expression: OPSCC CAFs clear of immune cell contamination. CAF1 and CAF2 were collected, and total RNA was extracted and quantified. qRT-PCR was performed to amplify the HLA-DR gene with β2m used for normalisation. MDM cells were used as positive control. CAFs culture exhibited a negligible expression of HLA-DR. ***p < 0.001, using one-way ANOVA. Error bars = SD of (N = 2, n = 3). (C) CD31 expression OPSCC CAFs cultures are clear of endothelial cell contamination. CAF1 and CAF2 were collected, and total RNA was extracted and quantified. qRT-PCR was performed to amplify the CD31 gene with β2m used for normalisation. HDMEC were used as positive control. CAFs culture exhibited a negligible expression of HDMEC. ***p < 0.001, using one-way ANOVA. Error bars = SD of (N = 2, n = 3). [file Image1.jpeg]

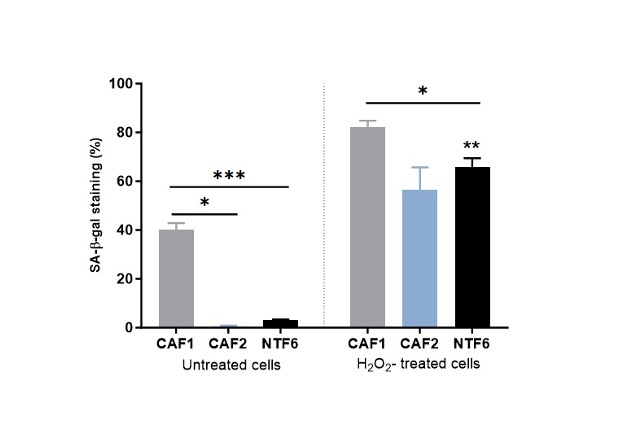

Supplement: Supplementary Figure S2 — Induction of senescence by H2O2 treatment in OPCAFs and NTF6. The three cultures were treated with 500mM H2O2 for 2h and maintained for 14 days to induce senescence. Quantification of the % of cells stained for SA-β-gal: data shows the mean proportion of cells stained blue in 3 random fields (magnification 20x). Confluent NTF6 were used as a positive control. *p < 0.05, **p < 0.01, ***p < 0.005. Error bars = SEM. [file Image2.jpeg]

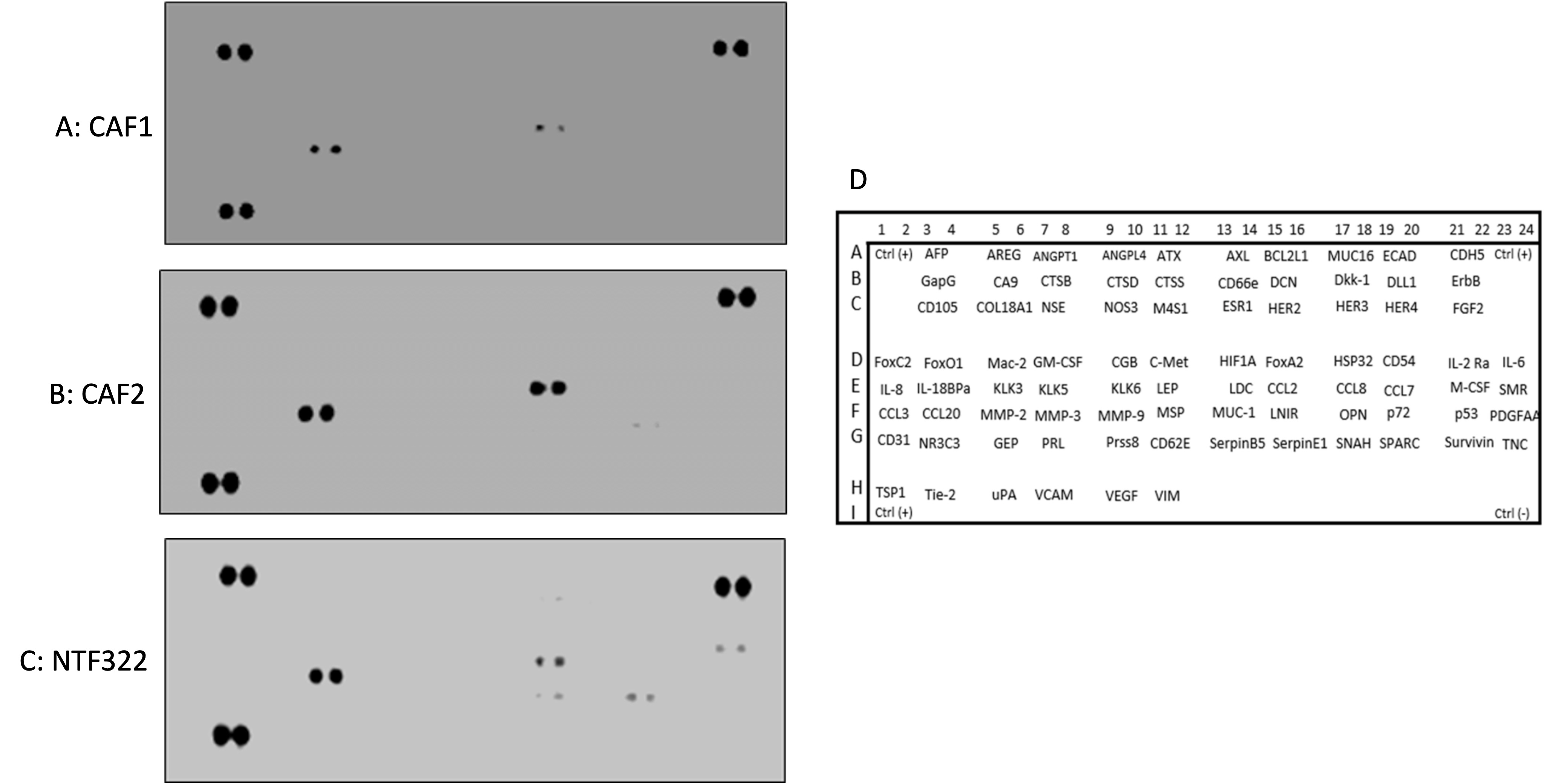

Supplement: Supplementary Figure S3 — Baseline CAF1, CAF2 and NFT322 cytokine production. Array images show the developed cytokine membrane for CAF media without treatment. The array images are (A) CAF1 CM: Only CCL2 and MMP2 were detected. (B) CAF2 CM: MMP2, CCL2 and SPARC (weak) were detected. (C) NTF322 CM: MMP2 spots showed strong expression, other cytokines (SPARC, Serpin B5, DCN) showed weak signals. The cytokine array map is also included (D). [file Image3.jpeg]

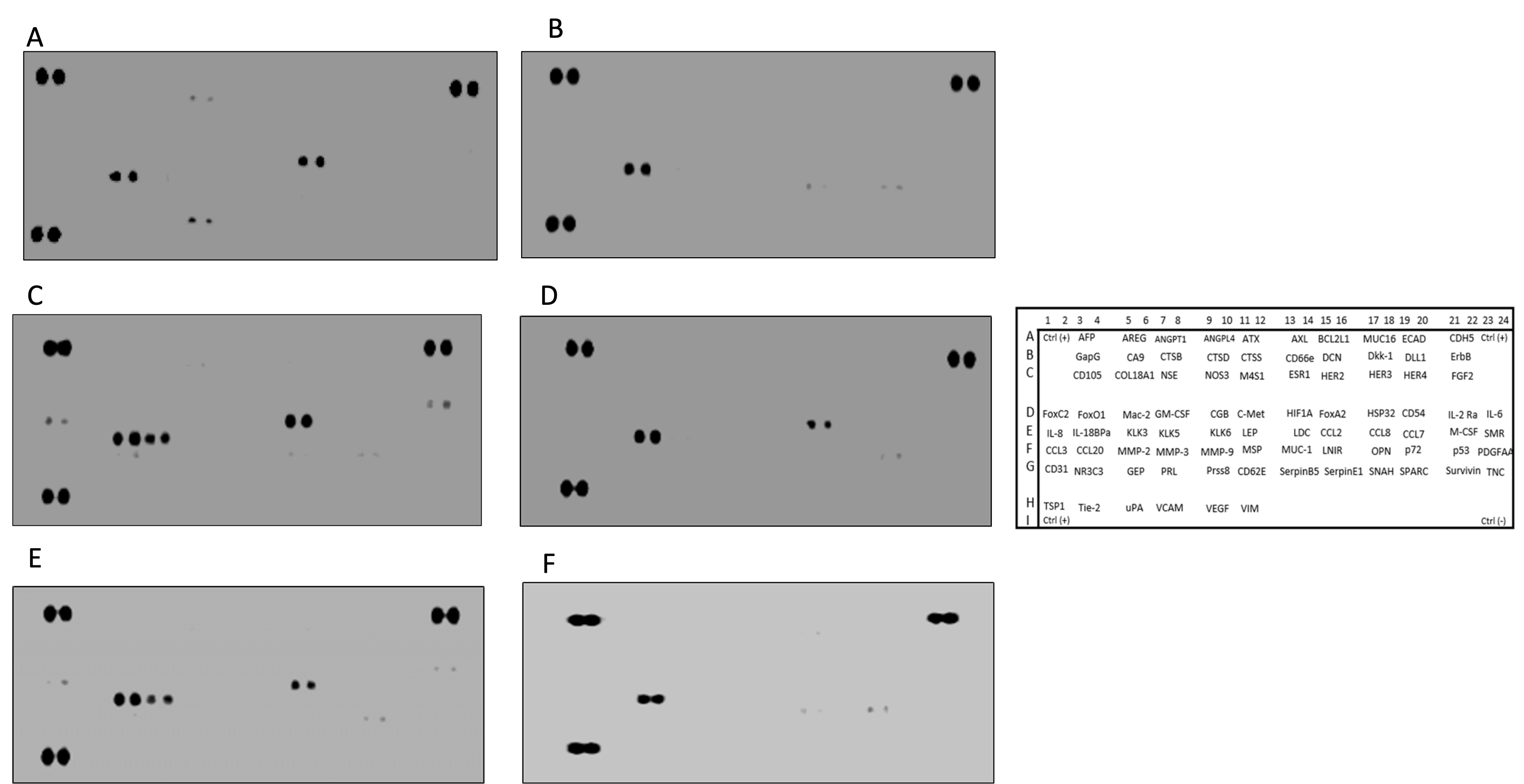

Supplement: Supplementary Figure S4 — Cytokine array analysis of CAF conditioned media, collected after application of fresh media, after the treatment of the fibroblasts with OPSCC CM for 24h. The array images are (A) CAF1 stimulated by SCC89 CM (B) CAF1 stimulated by SCC2 CM, (C) CAF2 stimulated by SCC89 CM, (D) CAF2 stimulated by SCC2 CM, (E) NTF322 stimulated by SCC89 CM, (F) NTF322 stimulated by SCC2 CM. The number of cytokines identified is reduced when compared to the combined CM presented in Figure 4, but represents the cytokines produced by the fibroblasts alone. Of note, a greater range of cytokines is present in the media from cells which have been treated by HPV-negative OPSCC CM (A, C and E). Of note, osteopontin (SPP1) is not identified, suggesting that it is produced by the SCC cells. [file Image4.jpeg]

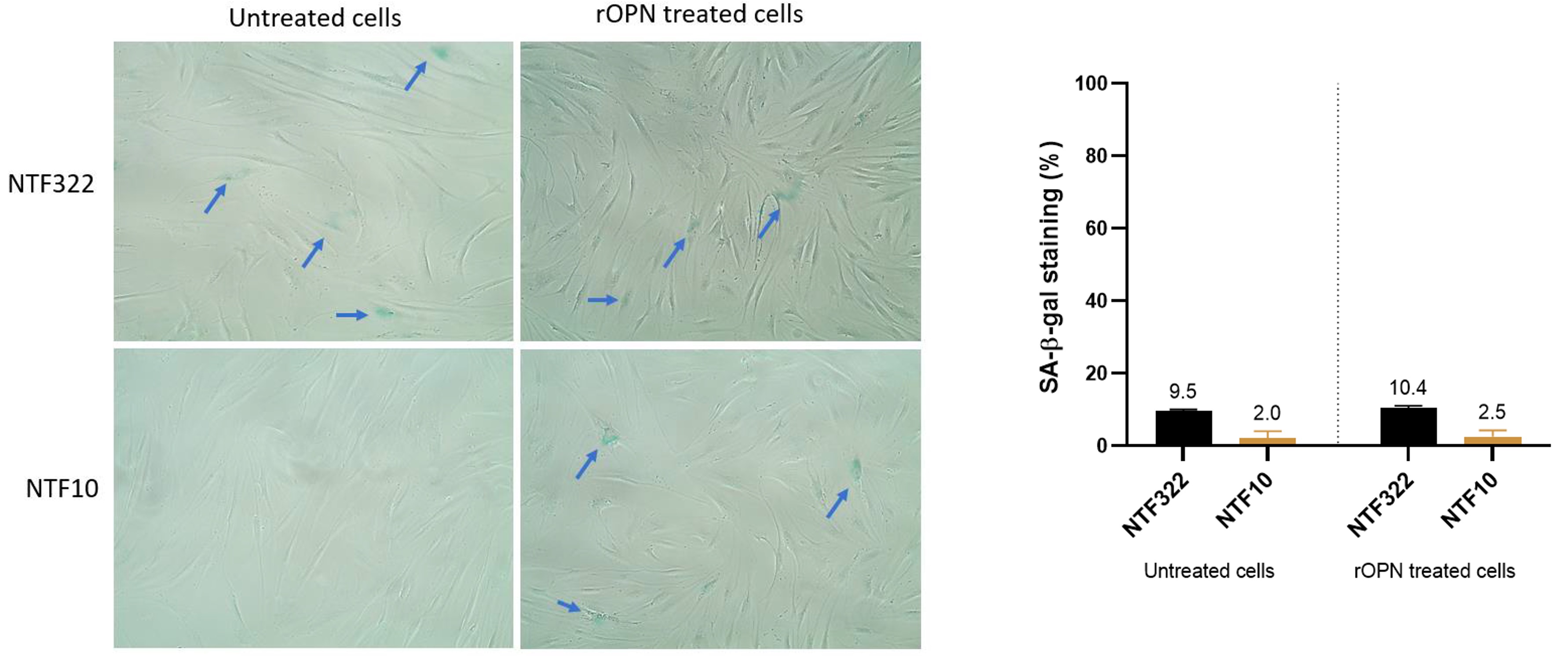

Supplement: Supplementary Figure S5 — Quantification of the proportion of senescent cells after rOPN treatment of NTFs. (A) Images representing SA-β-gal stain at basal level and after OPN treatment in NTF322 and NTF10, 10X magnification. (B) Quantification of SA-β-gal stain. The proportion of stained cells (blue precipitate) in 3 random fields at magnification 20x. Statistical analysis was performed using Student's t-test. Error bars = SD for (N = 3, n = 3). [file Image5.jpeg]
